# Review of Measures of Worksite Environmental and Policy Supports for Physical Activity and Healthy Eating

**DOI:** 10.5888/pcd12.140410

**Published:** 2015-05-07

**Authors:** J. Aaron Hipp, Dominic N. Reeds, Margaret A. van Bakergem, Christine M. Marx, Ross C. Brownson, Surya C. Pamulapati, Christine M. Hoehner

**Affiliations:** Author Affiliations: Dominic N. Reeds, Christine M. Marx, Washington University School of Medicine, St Louis, Missouri; Margaret A. van Bakergem, Vanderbilt University Medical Center, Nashville, Tennessee; Ross C. Brownson, Christine M. Hoehner, Prevention Research Center, Washington University in St Louis, St Louis, Missouri; Surya C. Pamulapati, University of Missouri, Columbia, Columbia, Missouri.

## Abstract

**Introduction:**

Obesity prevention strategies are needed that target multiple settings, including the worksite. The objective of this study was to assess the state of science concerning available measures of worksite environmental and policy supports for physical activity (PA) and healthy eating (HE).

**Methods:**

We searched multiple databases for instruments used to assess worksite environments and policies. Two commonly cited instruments developed by state public health departments were also included. Studies that were published from 1991 through 2013 in peer-reviewed publications and gray literature that discussed the development or use of these instruments were analyzed. Instrument administration mode and measurement properties were documented. Items were classified by general health topic, 5 domains of general worksite strategy, and 19 subdomains of worksite strategy specific to PA or HE. Characteristics of worksite measures were described including measurement properties, length, and administration mode, as well as frequencies of items by domain and subdomain.

**Results:**

Seventeen instruments met inclusion criteria (9 employee surveys, 5 manager surveys, 1 observational assessment, and 2 studies that used multiple administration modes). Fourteen instruments included reliability testing. More items were related to PA than HE. Most instruments (n = 10) lacked items in the internal social environment domain. The most common PA subdomains were exercise facilities and lockers/showers; the most common HE subdomain was healthy options/vending.

**Conclusion:**

This review highlights gaps in measurement of the worksite social environment. The findings provide a useful resource for researchers and practitioners and should inform future instrument development.

## Introduction

Overweight and obesity are major health challenges because of their high prevalence, causal relationship with serious medical complications, and economic impact (1). The risk of developing many diseases, including type 2 diabetes, increases linearly with body mass index (2–6). Obesity prevention strategies are needed that target multiple levels of the ecologic framework across multiple settings, including the worksite. Using the worksite as a venue for health promotion is promising, because most adults spend approximately half of their waking day in their work environment (6). Research suggests that environmental and policy strategies for addressing energy balance (ie, caloric intake and energy expenditure through physical activity [PA]) in the workplace are effective (7–9). Use of worksite programs to improve employee health has been recommended by the American Cancer Society, the Centers for Disease Control and Prevention, and multiple state governments. Occupational settings take advantage of a captive population and may have existing facilities, social support, convenience, and communication mechanisms in place (10).

Targeting work environments for energy balance includes using policies, programs, and organizational practices to influence behavior. Example work environments include onsite facilities such as gymnasiums, lockers, showers, accessible stairways, and healthy vending options. Policies and programs include subsidized external gymnasium memberships; incentives to bicycle, walk, or use public transportation for the commute to and from work; and group services such as onsite yoga and health fairs (11). By facilitating access to inexpensive healthy food, exercise facilities, and a culture accepting of nonsedentary work breaks, worksites can become sites for health promotion via a healthy energy balance (6). Although tools are available for assessing worksite environments and policies in place for PA and healthy eating (HE), no review has documented the content and measurement properties of these tools. Such a review of worksite energy measurement tools could serve as a guide for researchers, practitioners, and worksites in selecting among existing tools and understanding methodologic gaps to guide potential development of new instruments. The purpose of this review was to identify and assess the state of science concerning available measurement instruments related to worksite environment and policy supports for workplace energy balance.

## Methods

The literature review was completed in May 2014, using PubMed, OVID, MedLine, Web of Science, and the Registry of Measures from the National Collaborative on Childhood Obesity. We also searched sources of gray literature, including Google Scholar and state health departments. Search terms were key words for worksites, energy balance, and measurement: (work OR worksite OR workplace OR employer OR job) AND (physical activity OR physical fitness OR diet OR exercise OR obesity OR active commuting) AND (evaluation OR monitor* OR survey OR questionnaire OR data collection). Titles of applicable results were screened for their relevance to the assessment of worksite environment and policy measurement, tool development, and worksite interventions targeting PA and HE.

The search was restricted to articles published in English from 1991 through 2013. Abstracts were scanned and accepted if related to 1 or more of the following criteria designed to capture the presence or absence of worksite supports and policies associated with employee PA and HE (eg, presence of an onsite gymnasium, incentives to use public transportation to and from work): 1) studies describing measurement properties of a specific instrument, 2) descriptive studies of environmental and policy supports among a sample of employees or worksites, and 3) cross-sectional or intervention studies that used a specified instrument or explicitly stated the items used to systematically assess worksite environment and policies and their potential associations with PA and HE. Full-text articles were scanned when the information from abstracts was insufficient to make a conclusion about inclusion. Abstracts were excluded if they focused solely on the development or implementation or both of worksite health promotion programs and, thus, were not related to measuring current supports and policies. Moreover, abstracts were rejected if they did not emphasize policy or environmental supports in a nonhome-based worksite. Finally, full-text articles and their reference lists were scanned for references that cited the development of a specific worksite tool, survey, or checklist on policies and environmental supports related to PA and HE. The instruments used among articles that met inclusion criteria were abstracted. Each instrument was categorized on the basis of 1 of 4 administration options: employee or self-report, manager report, observational, or multiple modes. Measurement properties, including reliability and validity, were documented.

The final component of the review involved classifying each unique instrument item into an item inventory. Items were first classified by the *general health topic* they addressed: PA, HE, or both (healthy eating and physical activity [HEPA]). Next, items were classified by the general worksite strategy being assessed, referred to as the *primary domain*. These strategies are based on the ecological model, the *Guide to Community Preventive Services*, and research by Kahn et al (12,13) and include promotions and programs (eg, informational media), organizational policies and practices (eg, incentives), internal physical environment (eg, access to healthy food and PA options), internal social environment (eg, role models), and external environment (eg, worksite neighborhood options for HE and PA). Primary domains were further disaggregated into *subdomains* by using constant comparison to classify the PA (19 subdomains) and HE (19 subdomains) strategies (Table 1). Interrater agreement for classifying the instrument items was 85% among 3 raters.

**Table 1:** Physical Activity and Healthy Eating Domain Details, Review of Measures of Worksite Environmental and Policy Supports for Physical Activity and Healthy Eating, United States, 1991–2013

Subdomain	Description
**Physical Activity**	
**I. Promotion and programs**	**Key words: promote, posters, program, distribute**
Assessments/testing/evaluation	Employee fitness testing, measurements of employee PA, health screening
Counseling/classes/education	Informational support for participation in programs related to PA, organized PA activities (classes, clubs, long-term programs), and educational informative sessions (seminars, classes, meetings) that promote PA
Informational media	Worksite media sources or signage (posters, flyers, bulletin boards, maps) that encourage, promote, or direct employees to participate in active behaviors; sharing of information
**II. Organizational policies and practices**	**Key words: policy, guidelines, manager, worksite requirements**
Affordable options	Subsidies, worksite contributes financial assistance, free gymnasium access, insurance discounts
Time	Flex-time, specific policy where employees can participate in PA during work hours
Incentives	Worksite sponsors financial, material, or other types of prizes, incentives, and gifts for PA
Challenges	Worksite supports PA challenge (eg, steps per day)
Manager support	General statement about worksite, manager, or employer support or participation in PA initiatives
Community partnerships	Employer engages with entities outside of work environment; affiliating or collaborating with community organizations to improve health
**III. Internal physical environment**	**Key words: access, interior, facilities — anything indoors**
Access to PA equipment	Fitness centers, machines (ellipticals, treadmills), free weights, areas designated for PA
Stairway access	Access, visible, safe; general qualities about stairs
Lockers/showers	Access and availability; qualities about lockers/showers
Office connectivity	Hallways, passages, route, intersect, room, workstation
**IV. Internal social environment**	**Key words: coworker, support, values**
Role models for healthy choices	Peer modeling, coworkers as guides and good examples, coworker PA behavior
Coworkers’ support/encouragement	Positive interaction between employee and coworkers in favor of PA or healthy activities
**V. External physical and social environment**	**Key words: worksite neighborhood, outdoor, access**
Walkability	Land use mix, sidewalks/paths/trails, traffic, aesthetics, crime, safety, access to public transit
Parking (bicycle/vehicle)	Vehicle and bicycle outdoor parking, safe areas for bicycles, carpool parking spots, parking a vehicle farther away to increase walking distance to work
Active commuting/transit	Bicycle lanes, lockers, and showers only in reference to active commuting
Access to PA facilities	Walking distance to areas dedicated to PA, recreational facilities, parks, open space
**Healthy Eating**	
**I. Promotion and programs**	**Key words: promote, posters, program, distribute**
Assessments/testing/evaluation	Employee fitness testing, measurements of employee HE, health screening
Counseling/classes/education	Informational support for participation in programs related to HE, organized HE activities (classes, clubs, long-term programs), educational informative sessions (seminars, classes, meetings) that promote HE
Informational media	Worksite media sources or signage (posters, flyers, bulletin boards) that encourage, promote, or direct employees to participate in HE; sharing of information
**II. Organizational policies and practices**	**Key words: policy, written guidelines, manager, requirements**
Affordable options	Cafeteria has discounts for healthy food
Time	Flexible lunch breaks, sufficient time to eat properly, ability to leave work to access healthy food store, lunch is enforced at worksite
Incentives	Worksite sponsors financial, material, or other types of prizes, incentives, and gifts for HE
Healthy food at meetings/events	Specific to catered food, worksite contracts with healthy food service, provides fruits and vegetables and healthy beverages
Healthy options onsite/vending	Not presence of healthy food, but a policy for healthy alternatives in worksite cafeteria/vending; this includes specific polices that distinguish healthy items from nonhealthy items (ie, requirements for nutrition labeling) or those concerning food preparation and serving size. Or, manager/employer initiatives and efforts to offer healthy options
Manager support	General statement about worksite, manager, or employer support or participation in HE initiatives
**III. Internal physical environment**	**Key words: access, interior, facilities — anything indoors**
No-cost water	Water dispensers/coolers, drinking fountains, contracts with water company, available and free to employees at any time
Nutrition labeling	Presence of nutrition labeling in cafeteria or vending machines
Healthy options onsite/vending	Statement that healthy and nutritious options are available or offered onsite in both cafeteria and vending machines
Access to appliances	Worksite environment has access to refrigerator, microwave, toaster, or other appliances that make it possible for employees to bring food from home or cook during work
**IV. Internal social environment**	**Key words: coworker, support, values**
Healthy options for shared food	Birthdays, seminars, or activities where employees who bring food to share for social settings (not catered) are encouraged to be healthy or provide options for healthy treats/snacks
Role models for healthy choices	Peer modeling, coworkers as guides and good examples, coworker HE behavior, noticing that coworkers bring healthy lunches
Coworkers’ support/encouragement	Positive interaction between employee and coworkers in favor of HE or healthy activities
**V. External physical and social environment**	**Key words: neighborhood, restaurant, store, outdoor, access**
Access to healthy options	Not referencing a specific vendor (restaurant/store), but the availability of healthy foods not associated with a store/restaurant (eg, low-fat items, fruits and vegetables)
Types of food stores	Grocery stores, farmers market; stores where employees can shop for food
Types of restaurants/vending nearby	Fast food, convenience stores that sell food for immediate consumption

Abbreviations: HE, healthy eating; PA, Physical activity.

## Results

Seventeen worksite instruments were identified that included items about worksite environment and policies related to PA, HE, or both and met inclusion criteria. The administration modes of the 17 instruments varied (n = 9 self-report; n = 5 manager report, n = 1 observational; and n = 2 using multiple modes) as did the total number of HE and PA items per instrument (range, 10–226) (Table 2). More items were related to PA than to HE. Nine instruments included both PA and HE items, 7 instruments had only PA items, and only 1 included solely HE items related to worksite environment and policy supports. Of the 17 instruments, 14 reported reliability, of which 8 reported generally high interrater results (Table 2). Five instruments reported various validity measures including content, face, predictive, and construct validity results. Health promotion experts provided substantial guidance in development of the instruments, and significant correlations were found for workplace environmental sections within the instruments. The item inventory indicated that the most common health topic was PA (PA and HEPA) (64% of all items [n = 669]). HE (HE and HEPA) consisted of 369 items, or 36%.

**Table 2:** Worksite Questionnaire Details, Review of Measures of Worksite Environmental and Policy Supports for Physical Activity and Healthy Eating, United States, 1991–2013

Survey Name	Administration Mode	Year	Survey Details (No. of Items, Time Required)	Sample (a. Sample Size, b. Location, c. Type of Worksite)	Reliability	Validity	Health Topic
Worksite and Energy Balance Survey (WEBS) (19)	Self-report	2013	72, NR	a. 104 b. Missouri c. Variety	Test–retest by total population and by obesity status and size of worksite	NR	PA/HE
Office Environment and Sitting Scale (OFFESS) (20)	Self-report	2013	12, NR	a. 307 b. Australia c. Higher education campus	Internal consistency Test-retest % agreement overall and by office type	NR	PA
California Worksite Assessment Checklist (CA) (21)	Self-report	2010	31, NR	a. NA b. NA c. NA	NR	NR	PA/HE
(No Name) Kaczynski et al (22)	Self-report	2010	11, NR	a. 375 Full-time workers b. Manhattan, KS c. Variety	NR	NR	PA
Worksite Supportive Environments for Active Living Survey (SEALS) (23)	Self-report	2010	28, <30 min	a. 1,250 Working adults b. Mid-South United States c. Higher education campus	Internal consistency Test-retest Construct	Face Content Discriminant	PA
Check for Health (WI) (24)	Manager report	2010	68, NR	a. NA b. NA c. NA	NR	NR	PA/HE
Workplace Nutrition and Exercise Climate Scale (WNECS) (25)	Self-report	2010	119, NR	a. 156 Full-time workers b. Florida c. Variety	Internal consistency Interrater	NR	PA/HE
Environmental Perception Measure (EPM) (26)	Self-report	2009	10, <30 min	a. 23 Studies in literature review b. NA c. NA	Test–retest Internal consistency % Agreement	Predictive	PA
Community Healthy Living Index (CHLI) (27)	Manager report	2008	75, NR	a. Task force of 20 experts b. NA c. NA	Interrater	NR	PA/HE
Worksite Environmental Measure (WEM) (28)	Manager report	2007	105, >30 min	a. 4 Bus garages b. Minneapolis/St Paul c. Bus garage (indoor/outdoor)	Interrater	NR	PA/HE
Environmental Assessment Tool (EAT) (29)	Multiple	2006	105, >30 min	a. 12 Worksites b. Not reported c. Chemical companies	Interrater	Predictive Concurrent	PA/HE
Workplace Walkability Audit Tool (WWAT) (30)	Observational	2005	14, NR	a. 10 University campuses b. NA c. Higher education	Interrater	NR	PA
Neighborhood Quality of Life Survey (NQLS) (31)	Self-report	2004	32, NR	a. 1,313 Working adults b. Seattle, Baltimore, DC regions c. Not reported	Internal consistency	NR	PA
Workplace Physical Activity Framework (WPAF) (32)	Manager report	2003	45, 30 min	a. 15 Employees b. Alberta, Canada c. Education, municipality, hospital	Interrater	Content	PA
Working Well Trial (WWT) (33)	Self-report	1999	12, NR	a. 114 Worksites b. Massachusetts, Florida, National Cancer Institute	Internal consistency	NR	HE
Checklist of Health Promotion Environments at Worksites (CHEW) (34)	Multiple	1995	112, >30 min	a. 20 Worksites b. Australia c. Variety	Interrater	NR	PA/HE
Heart Check (HRTCHK) (35)	Manager report	1993	226, >30 min	a. >10,000 Employees b. New York c. Variety	Interrater internal consistency	Content face construct criterion	PA/HE

Abbreviations: HE, healthy eating; NA, not applicable; NR, not reported; PA, physical activity.

### Physical activity

**Figure 1:**
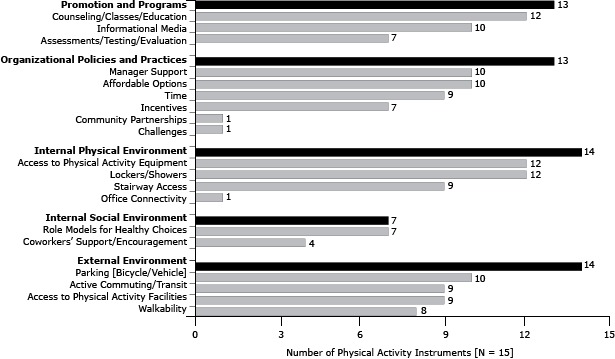
Number of instruments containing at least 1 item from each physical activity domain and subdomain (N = 15), review of measures of worksite environmental and policy supports for physical activity and healthy eating, United States, 1991–2013. **Table:** 

Two instruments, the Environmental Assessment Tool (EAT) (29) and the Checklist of Health Promotion Environments at Worksites (CHEW) (34), had the highest number of PA items (151 and 107, respectively) and used multiple modes of administration. Of the 17 instruments, only 1, Working Well Trial (WWT) (33), did not contain items related to PA. Of the surveys with PA items, most (14 of 16) included at least 1 item related to the external environment relevant for PA (Figure 1). The domain that was represented by the fewest number of instruments was the internal social environment, with only 7 total instruments containing at least 1 PA item for that domain. In terms of subdomains, only 1 instrument contained an item related to community partnerships, workplace challenges, or office connectivity, whereas 12 covered the subdomains counseling/classes/education, access to PA equipment, and lockers and showers.

Domain/Subdomain	No. of Instruments
**Promotion and programs**	**13**
Counseling/classes/education	12
Informational media	10
Assessments/testing/evaluation	7
**Organizational policies and practices**	**13**
Manager support	10
Affordable options	10
Time	9
Incentives	7
Community partnerships	1
Challenges	1
**Internal physical environment**	**14**
Access to physical activity equipment	12
Lockers/showers	12
Stairway access	9
Office connectivity	1
**Internal social environment**	**7**
Role models for healthy choices	7
Coworkers’ support/encouragement	4
**External environment**	**14**
Parking (bicycle/vehicle)	10
Active commuting/transit	9
Access to physical activity facilities	9
Walkability	8

Specific results for each instrument were also explored. Of the 19 subdomains for PA-related items, the California Worksite Assessment Checklist (CA) instrument included items covering the most subdomains (16 of 19 subdomains). The Workplace Walkability Audit Tool (WWAT) instrument covered the fewest subdomains (1 of 17 subdomains).

### Healthy eating

**Figure 2:**
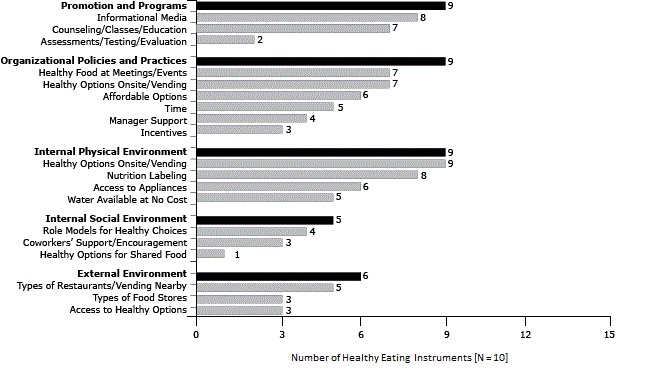
Number of instruments containing at least 1 item from each healthy eating domain and subdomain (N = 10), review of measures of worksite environmental and policy supports for physical activity and healthy eating, United States, 1991–2013. **Table:** 

Of the 5 primary domains, 3 (promotion and programs, organizational policies and practices, and internal physical environment) had the greatest coverage, with 9 of the 10 healthy eating instruments containing at least 1 item for each respective primary domain (Figure 2). Similar to the findings for PA domain coverage, the primary domain with the least coverage was the internal social environment; 5 of the 10 HE instruments covered that topic. Additionally, a noticeable gap is indicated through the external environment primary domain; only 6 instruments covered HE items related to the external food environment of worksites. The California Worksite Assessment Checklist (CA) instrument (21) spanned the greatest number of HE subdomains (15 of 19 subdomains). The HE instrument with the least coverage, Workplace Nutrition and Exercise Climate Scale (WNECS) (25), included items across 5 of the 19 subdomains.

Domain/Subdomain	No. of Instruments
**Promotion and Programs**	**9**
Informational media	8
Counseling/classes/education	7
Assessments/testing/evaluation	2
**Organizational Policies and Practices**	**9**
Healthy food at meetings/events	7
Healthy options onsite/vending	7
Affordable options	6
Time	5
Manager support	4
Incentives	3
**Internal Physical Environment**	**9**
Healthy options onsite/vending	9
Nutrition labeling	8
Access to appliances	6
Water available at no cost	5
**Internal Social Environment**	**5**
Role models for healthy choices	4
Coworkers’ support/encouragement	3
Healthy options for shared food	1
**External Environment**	**6**
Types of restaurants/vending nearby	5
Types of food stores	3
Access to healthy options	3

## Discussion

As a venue for delivering HE and PA efforts, worksites provide a channel for reaching the large segment of the population that is employed (147 million as of November 2014, according to the US Bureau of Labor Statistics) (6,10). Moreover, measuring environmental and policy supports for PA and HE in the workplace is an important component in assessing and addressing the factors related to overweight and obesity (14). This review of worksite measures identified various data collection instruments and highlights several matters that require further consideration and attention for future research.

The results of the item inventory highlight both extensive and deficient domain coverage for both PA- and HE-related items. Overall, the primary domains of promotion and programs, organizational policies and practices, and internal physical environment had the greatest coverage among HE and PA items. The primary domain of internal social environment had few items for either HE or PA. We also found several administration modes used, most instruments being self-report. Only 1 instrument was observational (WWAT), although several used multiple methods. With 14 of the 17 instruments relying on either employee or manager self-report, the state of worksite PA and HE measurement is susceptible to respondent and social desirability bias. Regarding measurement properties, most instruments (14 of 17) reported high reliability results, mostly interrater measures. Validity was assessed for 5 instruments, with emphasis on content validation.

There was variety in the content gaps of the measures reviewed. Overall, there were few documented measures about HE in and around the workplace. Most HE measures focused on onsite cafeteria and vending options but neglected external environments (eg, healthy options within a 10-minute walk), organizational policies (eg, healthy snacks at meetings and events), and the social environment. The promotion and programs domain contains 8 measures with items related to informational media and 7 with classes or education (both subdomains); however, only 2 of 10 instruments included any items on assessments, testing, evaluation, and HE. Provided that a full-time employee spends at least 8 hours per day at the worksite — therefore, at least 1 meal is consumed at or near work during most working days — the gaps in HE measures is an important finding that deserves further attention. Exploring the diverse aspects of food environments near workplaces, rather than solely assessing onsite cafeteria and vending options, would be beneficial.

Of the 5 domains, internal social environment was included in the fewest HE- and PA-related instruments. Social environments, including role models, champions, and support, are highly associated with PA and obesity (15,16). Among the subdomains, specialized instruments (ie, Office Environment and Sitting Scale [20], Kaczynski et al [22], and the WWAT [30]) had minimal, if any, coverage. Also, despite including more than 100 unique items, CHEW had minimal coverage for the HE subdomains (only 9 of 19 subdomains covered) (Appendix).

Performing this review did have challenges and limitations. Forcing instrument items into domains and especially subdomains presented some difficulties in operationalizing the specific items. Items could also fit into more than 1 subdomain. The process of developing the subdomains was iterative; new items forced ever greater specificity in the naming and operationalization of the 38 subdomains. However, the specificity of selected subdomains — such as walkability, which can include land use mix, aesthetics, and sidewalks, compared with stairway access, which only refers to the presence of stairs — still varies greatly. We were systematic and prescriptive in our literature search for worksite measures, but this may not be an exhaustive list of worksite instruments, especially those present in the gray literature. Finally, Carnethon and colleagues (17) suggest that efforts moving forward must not only focus on PA but also reduce sedentary behaviors at worksites, and this can be accomplished via policies and designs. Future worksite measurements must do a better job of including sedentary behaviors in their instruments.

This review provides a concise guide for employers to existing worksite measures on PA and HE, both for selecting appropriate assessment instruments for the worksite and as a means to introduce new policies and programs to support healthy workers. For example, employers can administer health risk appraisals in combination with organizational health promotion checklists that have been developed. This approach would provide information to the employee and employer where there may be overlap or gaps between worksite supports and health risks and benefits. Social and physical environments in and around the workplace should be designed to be conducive to recommended healthy behaviors (18). In addition, optimal environmental modifications should promote healthy behaviors while simultaneously minimizing the physical, organizational, and occupational risk in the work environment.

## Appendix

A. Supplemental figure. Breakdown of Worksite Instrument by Administration Mode, Review of Measures of Worksite Environmental and Policy Supports for Physical Activity and Healthy Eating, United States, 1991–2013. This file is available for download as a Microsoft Word document.

B. Supplemental figure. Subdomain Coverage by Instrument, Review of Measures of Worksite Environmental and Policy Supports for Physical Activity and Healthy Eating, United States, 1991–2013. This file is available for download as a Microsoft Word document.
