# Supplementary material for: Review of Measures of Worksite Environmental and Policy Supports for Physical Activity and Healthy Eating
Source: Prev Chronic Dis. 2015 May 7;12:E65. doi: 10.5888/pcd12.140410 (PMC4436045; doi:10.5888/pcd12.140410)
Supplement: Supplementary file 1 [file 14_0410_01.docx]

Appendix A. Breakdown of Worksite Instrument by Adminstration Mode
