# Supplementary figures and images for: Review of Measures of Worksite Environmental and Policy Supports for Physical Activity and Healthy Eating
Source: Prev Chronic Dis. 2015 May 7;12:E65. doi: 10.5888/pcd12.140410 (PMC4436045; doi:10.5888/pcd12.140410)

Appendix B. Sub-Domain Coverage by Instrument


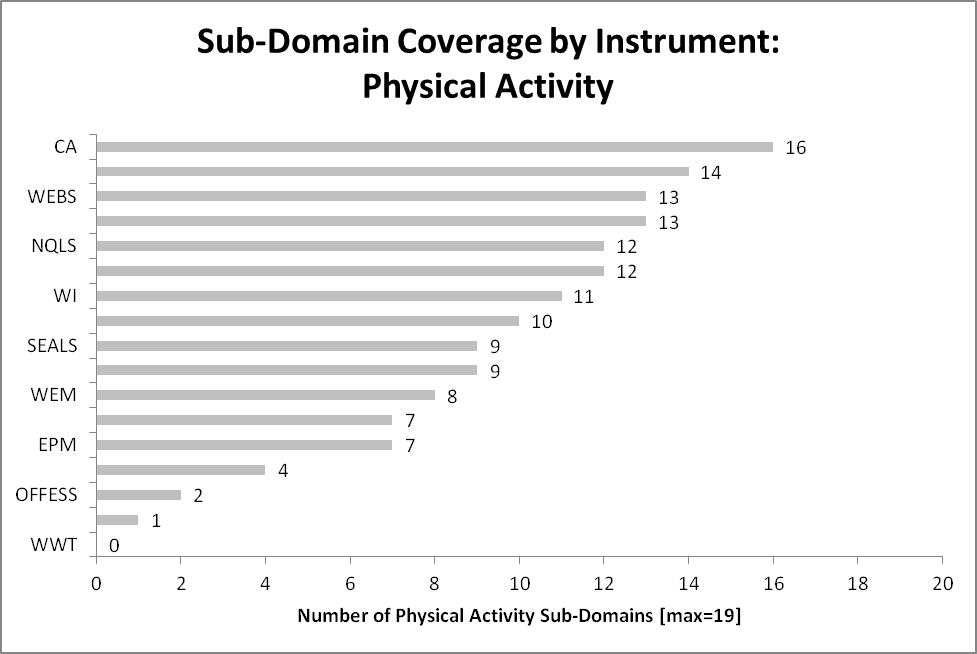

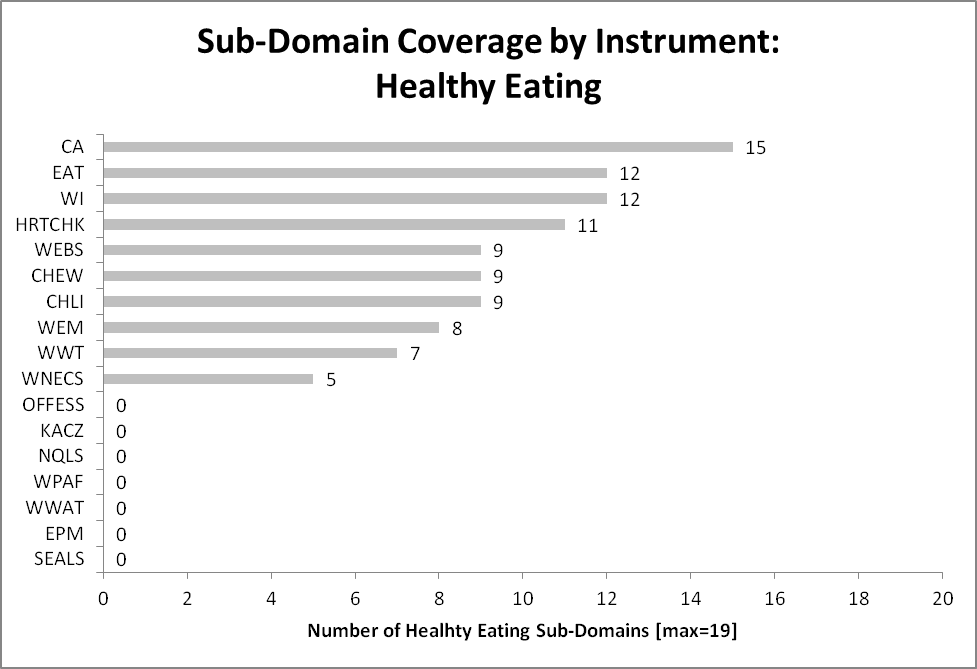

Supplement: Supplementary file 2 [file 14_0410_02.doc]
